# C-REGS 2 - Design and methodology of a high-quality comparative effectiveness observational trial

**DOI:** 10.25122/jml-2021-0362

**Published:** 2021

**Authors:** Johannes Vester, Natan Bornstein, Wolf-Dieter Heiss, Milan Vosko, Herbert Moessler, Marion Jech, Stefan Winter, Michael Brainin

**Affiliations:** 1. Department of Biometry and Clinical Research, idv Data Analysis and Study Planning, Krailling, Germany; 2. Department of Neurology, Shaare Zedek Medical Center, Tel Aviv, Israel; 3. Department of Neurology, Max Planck Institute for Metabolism Research, Koln, Germany; 4. Department of Neurology, Kepler University Hospital, Linz, Austria; 5. Comamo Life Sciences, Zell am Moos, Austria; 6. Department of Research and Development, Ever Neuro Pharma, Unterach, Austria; 7. Department of Clinical Neurosciences and Preventive Medicine, Danube University Krems, Krems, Austria

**Keywords:** cerebrolysin, high-quality comparative effectiveness, observational trial

## Abstract

The main aim of this study is to systematically record Cerebrolysin treatment modalities and concomitant medication, according to local standards, in patients with moderate to severe neurological deficits after acute ischemic stroke and to assess the impact of these parameters on therapy outcome during early rehabilitation (day 21) and on day 90. An open observational treatment design based on the principles of high-quality comparative effectiveness research (HQCER) has been chosen to capture the therapies as applied in real-world clinical practice. HQCER opens a new horizon for strengthening the validity of the results from observational trials, thereby enhancing the associated level of evidence. Rigorous pre-specification of analytical procedures and tight risk-based centralized monitoring were additional measures to improve the impact of the observational approach. The value for real-world studies has become obvious, and such studies based on comparative effectiveness designs supplement the classical study designs by enabling the inclusion of larger proband numbers and more statistical reliability for practical use.

## Introduction

### Background information

#### Methodological Considerations

Observational studies are best used to evaluate the real-world applicability of evidence derived from randomized trials [1]. However, in terms of rating quality of evidence, observational trials usually begin as low-quality evidence, limiting the validity and generalizability of the observational results GRADE Guidelines *Rating the quality of evidence* [2, 3]. Recent suggestions for high-quality comparative effectiveness (CE) research GRACE principles for *Good Research for Comparative Effectiveness OBSERVED* [4, 5], in combination with rigorous risk-based centralized monitoring approaches (see EMA [6], FDA [7] risk-based guidance) open new perspectives for enhancing the level of evidence of observational trials.

Thus, in agreement with current recommendations, the present design of an observational registry study has chosen a high-quality comparative effectiveness approach.

#### Treatment

Cerebrolysin is a neuropeptide preparation with marketing authorization to treat cerebrovascular disorders and neurodegenerative diseases for many years worldwide. Since its first approval, stroke therapy has developed, and new treatment concepts have been implemented. In addition, over time, Cerebrolysin treatment in stroke has evolved with different time windows, dosages, and duration of therapy, often being given pragmatically by physicians. Therefore, the main aim of this study is to capture these variables of Cerebrolysin treatment and its comedication in patients with moderate to severe neurological deficits after acute ischemic stroke to guide further research.

C-REGS 2 is an international, non-interventional, prospective registry study to observe clinical practices of routine use of Cerebrolysin in patients with moderate to severe neurological deficits after acute ischemic stroke in a controlled and open-label manner. According to local treatment standards, all patients receive acute stroke care, which is not amended or influenced by the study. The outcome of Cerebrolysin-treated patients is compared with control group patients, who do not receive Cerebrolysin to evaluate the safety and effectiveness of Cerebrolysin in routine practice.

## Material and Methods

### Study Objectives

The objective of this registry study was to observe clinical practices, safety, and effectiveness of routine use of Cerebrolysin in the treatment of patients with moderate to severe neurological deficits after acute ischemic stroke, based on principles of high-quality comparative effectiveness research (HQCER).

### Study Design

Non-interventional, controlled, open-label, prospective, multicentre, high-quality comparative effectiveness observational registry study.

### Patient population

Criteria for observed patient population:

• Signed Informed Consent;
• Clinical diagnosis of acute ischemic stroke, confirmed by imaging;
• Moderate to severe neurological deficits (observation window: NIHSS 8-15);
• No prior stroke;
• No prior disability;
• Patient’s independence before stroke onset (premorbid mRS of 0 or 1);
• Reasonable expectation of successful follow-up (max. 100 days).

### Investigational Product

Cerebrolysin has marketing authorization in the countries participating in C-REGS 2. C-REGS 2 is an observational study. Thus, the treatment of patients follows standard hospital practices and is separated from the decision to include a patient in the study.

#### Name and Description of the Investigational Product

Cerebrolysin is a neuropeptide preparation produced by a standardized enzymatic breakdown of purified, lipid-free porcine brain proteins. It consists of low molecular weight neuropeptides (<10 kDa) and free amino acids. Cerebrolysin has been used to treat cerebrovascular and neurodegenerative diseases, including stroke, dementia, and traumatic brain injury, for many years.

#### Dosage, Formulations, and Administration

Dosage, frequency, duration, and mode of administration of Cerebrolysin follow the local hospital practice following the terms of the local marketing authorization and are not amended or influenced by the study. Prescribed Cerebrolysin will be used as a solution for injection/concentrate for solution for infusion.

### Concomitant Therapy

Concomitant medication is not restricted or influenced by the study and is documented in the eCRF.

### Assessment of Effectiveness

No additional diagnostic, therapeutic, or monitoring procedures other than those used according to local practice will be applied to the patients included in the study. Tests selected to assess effectiveness have been chosen per the recommendations of various stroke guidelines:

The *Modified Rankin Scale* [8] (mRS) measures the level of disability after stroke and has been widely used for many years in clinical practice to measure global functional outcomes.

The mRS assessment is performed using the Rankin Focused Assessment (RFA) [9, 10] to provide clear operationalization and ensure consistent score determination across raters.

The *National Institutes of Health Stroke Scale* (NIHSS) is a systematic assessment tool that provides a quantitative measure of stroke-related neurological deficits. It is described as the standard for the assessment in acute stroke in the “Guidelines for the early management of patients with acute ischemic stroke” by the American Heart Association/American Stroke Association, the ESO Guidelines for “Management of Ischemic Stroke and Transient ischemic Attack” and in the Austrian Neurology Society (ÖGN-ÖGSF) guideline (Chapter 12) [10, 11].

The NIHSS reflects neurological impairment, the clinical domain in which early effects of acute stroke therapies are likely to be most marked. Recent research showed that the NIHSS is most sensitive for early points in time [12]. Furthermore, it is less influenced by extraneous factors, improving sensitivity to acute treatment effects. A trained observer rates the patient’s ability to answer questions and perform activities. Ratings for each item are scored with 3 to 5 grades with 0 as normal, and there is an allowance for untestable items. The single patient assessment requires less than 10 minutes.

The assessment of cognitive deficits following stroke is recommended by several guidelines (ESO Guidelines, Austrian Neurology Society [13] and the DEGAM Stroke Guideline nr. 8). The DEGAM guideline mentions the Mini-Mental State Examination (MMSE) for assessment. However, compared to the MMSE, the *Montreal Cognitive Assessment* (MoCA) was shown to be more sensitive for the detection of cognitive impairment after acute stroke [14] and is recommended by the “National clinical guideline for Stroke” [15]. The MoCA is a screening tool for mild cognitive dysfunction, which takes approximately 10 minutes [16]. The possible maximum score is 30 points, and 26 or above is considered normal.

The *Informant Questionnaire on Cognitive Decline in the Elderly* (IQCODE) is described as a valuable tool to assess premorbid cognitive dementia [17], which has been widely adopted in clinical practice [18]. The IQCODE is a prerequisite for the MoCA assessment to identify patients with cognitive dysfunction before a stroke. It is filled out by a person who has known the patient for ten or more years.

#### Mandatory Training of Scales

For the NIHSS, the mRS, and the MoCA, mandatory training has to be performed by all personnel using the scales. The rating has to be performed by site personnel that is independent of treatment administration.

### Statistical Methods

#### General Principles

Selection of patients for exposure to treatment based on clinical features and physician preference instead of random allocation inevitably introduces opportunities for bias and confounding. According to the principles of Good Research for Comparative Effectiveness Research (GRACE) [4] and in line with the HTA recommendations for non-randomized studies [19], appropriate control of confounding variables and pre-specification of analytical techniques is implemented as one of the primary requirements for high-quality comparative effectiveness research. In addition, rigorous risk-based centralized monitoring is introduced to enhance the validity of the observational trial further.

#### Effectiveness Evaluation

Ordinal analysis of the modified Rankin scale (mRS) three months after stroke onset is chosen as a clinically relevant primary endpoint for final treatment effects. The leading secondary endpoint is the NIHSS score on days 21 and 90.

The technical operationalizations for the first-line analysis of the primary and secondary effectiveness measures, based on observed cases (OC) and target population (see section Analysis Sets), are as follows:

• Primary outcome measures (effectiveness evaluation)
• Secondary outcome measures (effectiveness evaluation)

1. Ordinal modified Rankin Scale (mRS) at 3 months after stroke onset, absolute scores, Mann-Whitney Effect Size (MW) [20–25], OC, Target Population;
2. Ordinal NIH Stroke Scale (NIHSS) at 21 days and 3 months after stroke onset, absolute scores, Mann-Whitney Effect Size (MW), OC, Target Population;
3. Ordinal modified Rankin Scale (mRS) at 21 days after stroke onset, absolute scores, Mann-Whitney Effect Size (MW), OC, Target Population;
4. Proportion of patients with excellent recovery (mRS score 0–1) at three months after stroke onset, Odds Ratio, OC, Target Population;
5. Proportion of patients with functional independence (mRS score 0–2) at three months after stroke onset, Odds Ratio, OC, Target Population;
6. Ordinal MoCA at 3 months after stroke, Mann-Whitney Effect Size (MW), OC, Target Population.

According to the ICH Guideline E9 (ICH Topic E9, Statistical Principles for Clinical Trials, Step 4, Consensus guideline, 5 February 1998, CPMP/ICH/363/96) the results will be given as P-values as well as effect size measures with their associated confidence intervals (outcome no. 1, 2, 3, 6: Mann-Whitney effect size; outcome no. 4 and 5: odds ratio, supplemented by Mann-Whitney effect size for inter-outcome comparisons [25]), so that the direction and quantity of the treatment effects are determined with their precision.

The Mann-Whitney effect size [20–25] is the most valuable effect size measure for nonparametric approaches based on the well-known Wilcoxon framework because it is valid in data situations where the Hodges-Lehmann shift parameter is no longer appropriate. Furthermore, the Mann-Whitney effect size is appropriate for continuous, ordinal, and binary data at the same time and represents an ideal effect size measure. Incidentally, the 25^th^ Anniversary of the journal Statistics in Medicine dedicated a whole issue to papers about the Mann-Whitney statistic [20].

The Mann-Whitney effect size measure (MW) gives the probability that a randomly chosen subject of the test group is better off than a randomly chosen subject of the comparison group, defined in statistical shortcut: P (X<Y) + 0.5 P (X=Y). The null and alternative hypothesis for the comparison of Cerebrolysin vs. placebo can be formulated as follows (superiority test) by applying the Mann-Whitney effect size measure:



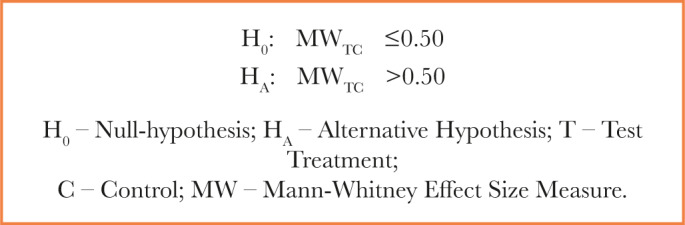



The traditional benchmarks for the Mann-Whitney effect size measure (MW) are as follows [26, 27]:



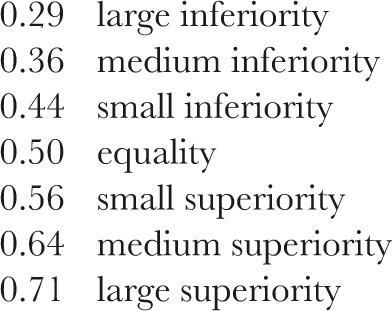



The global alpha of the trial is 0.05 two-sided. The primary outcome measure will be analyzed according to the pre-defined Bauer-Koehne alpha for stage I of the trial (see section 0). However, the secondary outcomes will be analyzed using the same alpha, applying the principle of a *priori* ordered hypotheses (fixed sequence) for multiplicity control. If the test for superiority concerning the primary outcome measure shows statistical significance, the secondary criteria can be tested with the same alpha as the first test with complete control of the study-wise type I error. The sequence and nature of the a *priori* ordered test-statistical hypotheses are defined above (outcome measures no. 1–6). The procedure of a *priori* ordered hypotheses is most potent with full control of alpha (for control of alpha using stepwise testing see [28]).

#### Safety Evaluation

The operationalizations for the evaluation of the pre-defined safety measures, based on observed cases (OC) and intention-to-include (ITI) population, are as follows:

1. Mortality, Odds Ratio, OC, Target Population;
2. Serious Adverse Events, Odds Ratio, OC, Target Population;
3. Adverse Events, Odds Ratio, OC, Target Population.

These safety measures will be used for group comparisons. In addition to the Target Population analysis with control of confounders, sensitivity analyses will be performed based on the ITI population. Adverse drug reactions to Cerebrolysin (ADR), serious adverse drug reactions to Cerebrolysin (SADR), and suspected unexpected serious adverse reactions to Cerebrolysin (SUSAR) will be displayed for the Cerebrolysin treatment group.

#### Case-Mix Standardization

The patient groups are standardized using nonparametric multilevel stratification procedures combined with a “restricted cohort” design to minimize enrollment bias. The individual risk factors have been identified from previous research results on NIHSS predictor variables, allowing appropriate control for confounders of outcome after acute ischemic stroke. The pre-specified case-mix standardization strategy follows the recommendations of the GRACE Principles for Good Research on Comparative Effectiveness [4, 5].

Pre-Defined Clinical Predictor Variables [29]:

1. Initial NIHSS;
2. Small Vessel Disease (yes-no);
3. Prior Stroke (yes-no);
4. Prior Diabetes (yes-no);
5. Prior disability (yes-no);
6. Age.

The operational definitions of variables no. 2 to 6 for consistent application across all participating sites are provided in the operational manual of the trial. The combination of the above variables is a highly efficient predictor for outcome after ischemic stroke [29], making an additional control of infarct volume dispensable due to comparable areas under the receiver operator characteristic (ROC) curves [29].

**Figure 1.**
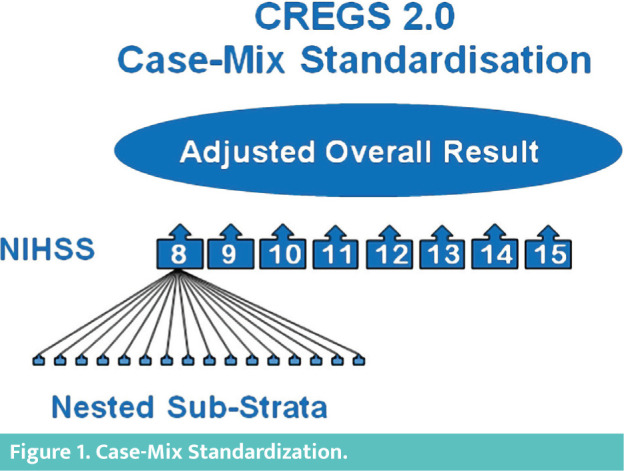
Case-Mix Standardization.

The top-level case-mix standardization is based on the initial NIHSS score as one of the strongest predictors for outcome after stroke [29–31]. The top-level control is performed by implementing stratification per NIHSS score unit with subsequent meta-analytic pooling of strata (i.e., comparing groups within identical baseline NIHSS score) (Figure 1). The eligibility restriction to NIHSS 8-15 allows full stratification for each possible baseline score (leading to a total of eight top-level strata).

The pre-planned method for synthesis of the strata based on the primary Mann-Whitney (MW) effect size measure [20–25] is the Wei-Lachin test of stochastic ordering (one-dimensional test) [32], a maximin-efficient robust test (MERT) [33, 34] which provides a combined MW estimate and test of overall treatment effect from the pre-defined ensemble of independent strata.

The second level case-mix standardization is performed to control further confounders (see pre-defined clinical predictor variables). It is implemented within each of the top-level NIHSS strata by means of nested sub-strata and subsequent adjustment through the Cochran-Mantel-Haenszel (CMH) pooling procedure (also known as the van-Elteren procedure) [35]. The nested sub-strata are based on the following pre-defined clinical predictor variables (see above):

• Diabetes (yes-no);
• Small Vessel Disease (yes-no);
• Age (<65 – ≥65 years).

The combination of the three binary predictor variables results in a total of eight nested sub-strata. Technically the robust Peto-Wilcoxon test with CMH pooling of sub-strata was chosen, providing adjusted MW across sub-strata with associated confidence intervals. This procedure allows unbiased adjustment of ordinal or binary data also in the presence of very low sample sizes (only sub-strata with total N<3 are excluded from CMH analysis).

In addition to the specified multilevel case-mix standardization, controlling four out of the six pre-defined predictor variables, a specific method to strengthen observational, non-interventional studies is introduced for control of the two remaining confounders (“prior stroke” and “prior disability”): the “restricted cohort” design, i.e., patients are only eligible for this trial without prior stroke and prior disability. Restricted cohort design is one of the measures recommended for high-quality comparative effectiveness research [19]. This way, any risk of bias associated with the specific confounders can be avoided.

The described case-mix standardization for control of confounders is performed for all comparative effectiveness evaluations, resulting in an adjusted overall effect size with an associated confidence interval for each defined endpoint. The same applies to the comparative safety evaluations to minimize confounding (see, e.g., FDA Guidance to Industry on Reporting Safety Studies) [36]. Unadjusted safety analyses, including all available patient data, will be performed as additional sensitivity analysis.

#### Two-Stage Procedure

The two-stage adaptive procedure of Bauer P and Köhne K (1994) is chosen as the sequential method. The two-stage procedure based on Fisher’s combination test (Bauer and Köhne) shows only a negligible loss in test power as compared to a fixed sample size study but allows early stopping due to success or failure [37]. Furthermore, assumptions for sample size calculation can be rechecked after stage I. The same applies to design modifications within the framework of the adaptive approach, although this is not the rationale for introducing the two-stage procedure in this study. The formal Bauer-Köhne *futility* benchmark is set for this study to α_0_=0.3. It is important to note that this benchmark takes into account the limited number of available patients for a possible stage II due to the restricted cohort design.

With a global multiple level alpha=0.05 two-sided, and defined futility level of α_0_=0.3 the following decision structure will be formally established (p_1_=P-value of stage I, p_2_=P-value of stage II):

Decision Structure for Stage I results (two-sided)

**Table:** 

p_1_≥α_0_=0.3	: stop because of futility
p_1_ϵ (0.0299; 0.3)	: continue with stage II
p_1_≤α_1_=0.0299	: stop with success (rejection of H_0_)

Decision Structure for Stage II results (two-sided)

**Table:** 

p_1_p_2_>α_c_=0.0087	: stop because of futility
p_1_p_2_≤α_c_	: rejection of H_o_ (proof of efficacy)

### Sample Size

As this is an observational study, the relations among effect size, sample size and power estimates are indicative only. However, some justification is needed to support the planning of the number of centers and the duration of the enrolment period.

Formal nonparametric sample size calculation was performed to allow detection of “small” group differences in the ordinal comparative effectiveness evaluations with 90% power.

The sample size calculations are based on the following design specifications:

a. Two-sided type I error defined as alpha=0.05 (multiple level alpha);
b. 90% power (1 – beta);
c. Effect size measure for ordinal scales: Mann-Whitney measure of superiority (MW) [20–25, 38–40];
d. Difference to be detected: MW=0.55 (equivalent to a “small” difference according to Cohen [26, 27]);
e. Assumed maximum imbalance between enrolled groups: 1:2;
f. Two-Stage Adaptive Design with α_0_=0.3; Stage I sample size=30% of total sample size (r_subsample I_=0.3); power compensation for defined two-stage parameters based on Newton Cotes-algorithm of fifth order [41];
g. Nonparametric model: stochastic superiority (minimized assumptions).

In the presence of the above assumptions, the calculated total sample size results in 1745 subjects (including power adjustment for the chosen parameters of the two-stage procedure). For compensation of usual ambiguities (dropouts etc), the calculated sample size is enhanced by a factor of 1.15 (15%) from 1745 to a total of approx. 2000 subjects. This way, at least 90% power is guaranteed within the framework of the two-stage procedure.

Stage I is completed after enrollment of about 30% of the planned total patients (r_subsample I_=0.3).

Nonparametric sample size calculations within the framework of a Two-Stage procedure (Bauer-Köhne) [41] were based on the model of stochastic superiority and have been performed applying the validated software Nnpar 1.0 and Bauer-Köhne 4.0 from IDV Data Analysis and Study Planning, Krailling/Munich).

### Risk-Based Centralized Statistical Monitoring

Data will be captured using an eCRF system with quality assurance performed by edit checks and frontline risk-based control. In addition, and in order to comply with recent calls for high-quality non-interventional comparative effectiveness research [5], an independent risk-based centralized statistical monitoring is introduced in combination with targeted on-site monitoring for ongoing surveillance of study conduct, thus ensuring the highest standards of data quality and integrity according to the most recent requirements of the ICH E6 Guideline for Good Clinical Practice (GCP, Amendment R2, July 2015) [42], the FDA Guidance for Industry on a Risk-based Approach to Monitoring [7], and the EMA reflection-paper on Risk-based Quality Management in Clinical Trials [6].

### Prospective Meta-Analysis (PMA)

Meta-analytic techniques are recognized as a useful tool to summarize the overall efficacy results of a drug application (ICH E9 biostatistical guideline, CPMP/EWP/2330/99). An extension of this approach is a prospective meta-analysis (PMA) in which studies are identified, evaluated, and determined to be eligible before the results of any of the studies become known [43] (see also Prospective Meta-analysis, Cochrane Handbook for Systematic Reviews of Interventions; Part 3, Chapter 19. http://handbook.cochrane.org).

Two registry trials on Cerebrolysin after stroke are currently implemented: the HQCER trial C-REGS 2, and the [Non-HQCER] trial CREGS-S, both with similar endpoints and a 90-day follow-up:

• C-REGS 2 (EVER-AT-0717);
• CREGS-S (EVER-GB-0514).

After completing both studies, a meta-analytic combination of these two registry trials was regarded as a useful complement to the individual study analyses and pre-specified in the final C-REGS2 statistical analysis plan (SAP Version Final 1.0 from 24 October 2017) before enrollment of the first patient.

Thus, according to the final SAP and after the termination of both trials, the present C-REGS 2 registry trial data will be combined with the data of the CREGS-S registry trial by formal meta-analysis procedures to gain further insight into the effectiveness of Cerebrolysin after stroke. As pre-specified in the final SAP and for ensuring consistent analysis data, the statistical operationalizations for the HQCER trial C-REGS 2 will be equally applied for the CREGS-S data. This is achieved by using individual patient (IPD) data analysis of both trials, the gold standard for meta-analytic pooling [44].

The technical sequence is as follows: in a first step, the Individual Patient Data (IPD) analysis of the trial CREGS-S will be executed according to the associated pre-specifications in the C-REGS 2 statistical analysis plan (SAP Version Final 1.0 from 24^th^ of October 2017). In a second step, the IPD analysis of the C-REGS 2 trial is performed after completion. In a final step, the results of the two preparational IPD analyses will be combined by formal meta-analysis. The meta-analysis will be conducted on the following endpoints (C-REGS 2 SAP operationalization):

• Primary outcome measures (effectiveness evaluation)

1. Ordinal modified Rankin Scale (mRS) at 3 months after stroke onset, absolute scores, Mann-Whitney Effect Size (MW) [20–25], OC, Target Population as operationalized in the C-REGS 2 SAP.

• Secondary outcome measures (effectiveness evaluation)

1. Ordinal NIH Stroke Scale (NIHSS) at 3 months after stroke onset, absolute scores, Mann-Whitney Effect Size (MW), OC, Target Population as operationalized in the C-REGS 2 SAP;
2. Proportion of patients with excellent recovery (mRS score 0-1) at 3 months after stroke onset, Odds Ratio, OC, Target Population as operationalized in the C-REGS 2 SAP;
3. Proportion of patients with functional independence (mRS score 0–2) at 3 months after stroke onset, Odds Ratio, OC, Target Population as operationalized in the C-REGS 2 SAP;
4. Ordinal MoCA at 3 months after stroke, Mann-Whitney Effect Size (MW), OC, Target Population as operationalized in the C-REGS 2 SAP.

These endpoints are identical to this SAP’s definitions for C-REGS 2, except Day 21 endpoints, since the CREGS-S trial does not involve any Day 21 assessments.

The pre-planned method of synthesis for the primary Mann-Whitney (MW) effect size measure [20–25] is the Wei-Lachin test of stochastic ordering (one-dimensional test) [32], a maximin-efficient robust test (MERT) [33, 34] which provides a combined MW estimate and test of overall treatment effect from an ensemble of independent studies. This approach is assumption-free and is robust also concerning the presence of heterogeneity [32]. Qualitative interaction will be tested using the Gail-Simon test [45], with P-values <0.10 preventing formal combination of studies.

As sensitivity analysis, the “classic” approaches based on fixed-effects model (Hedges-Olkin) [46] and random-effects model (DerSimonian-Laird) [47] will be calculated. Associated tests for quantitative heterogeneity will be performed using standard chi-square statistic [48] and I^2^ statistic [49]. The meta-analyses will be performed using the software packages METASUB (Version 4.1), and ForestPlot (Version 4.1) from IDV Data Analysis and Study Planning (Krailling/Munich, Germany) on high-security PCs (HSPC) within a validated working environment.

## Discussion

Selection of patients for exposure to treatment based on clinical features and physician preference instead of random allocation inevitably introduces opportunities for bias and confounding. Thus, according to Good Research for Comparative Effectiveness Research (GRACE) principles, and in line with the HTA recommendations for non-randomized studies, we placed great value on appropriate control of confounding variables together with pre-specification of all analytical techniques. Other important features were the consistent selection of analytical techniques with minimized assumptions and rigorous risk-based centralized monitoring to enhance the quality of the observational trial further.

An important decision for optimized control of confounding factors was the choice of a restricted NIHSS inclusion window – initial NIHSS score being one of the strongest predictors for outcome after stroke. Earlier randomized clinical studies on Cerebrolysin suggested reduced efficacy in patients with mild ischemic stroke [50]. De Graba *et al.* [30] underlined the significant implication of this benchmark in treatment protocols, highlighting that 45% of the patients with an initial score ≤7 were functionally normal already at 48 hours after acute stroke. Following these findings, we decided to restrict the lower inclusion benchmark to an NIHSS score of 8 – thus focusing on *moderate-severe* stroke severity. A recent meta-analysis on nine Cerebrolysin trials [51] confirmed this decision with rather weak effect sizes in mild stroke patients. To note that also the most recent joint EAN/EFNR guideline on pharmacological support in early motor rehabilitation after ischemic stroke [52] provided a recommendation for Cerebrolysin limited to moderate to severe ischemic stroke. As opposed to the lower NIHSS benchmark, the choice of the upper NIHSS limit was due to technical requirements of the defined multilevel case-mix standardization, allowing this way to fully control stroke severity across all eight single NIHSS strata of the target population (score between 8–15).

The advantage of the chosen multilevel case-mix standardization compared to other, model-based approaches such as regression models is that any assumption about the nature of risk-outcome relation is avoided, allowing true *like-to-like* comparisons. Furthermore, some potential drawbacks of other procedures, as the model-based propensity score matching with its risk of bias due to incomplete matching [53], are reduced.

According to the Principles for Good Research on Comparative Effectiveness (GRACE) recommendations, the study size intended for the observational trial “should be described including a description of how that size was determined, what specific assumptions are being made, and how well these assumptions are supported”. As this is an observational study, the relations among effect size, sample size, and power estimates are indicative only. However, some justification is needed to support the planning of the number of centers and the duration of the enrolment period. Thus, within the framework of the chosen study design, we performed a fully elaborated sample size calculation based on the pre-specification of the analytical techniques.

## Conclusion

High-quality comparative effectiveness research (HQCER) opens a new horizon for strengthening the validity of the results from observational trials and enhancing the associated level of evidence. In combination with rigorous pre-specification of analytical procedures and implementation of tight approaches to risk-based centralized monitoring, the impact of real-world observations may substantially profit from methodological improvements. The C-REGS 2 trial on safety and effectiveness of routine use of Cerebrolysin in the treatment of patients with moderate to severe neurological deficits after acute ischemic stroke introduces a bundle of quality-related HQCER measures, hopefully triggering similar approaches also in other observational studies and opening the awareness for methodologies options nowadays available to achieve this goal.

## Acknowledgements

### Conflict of interest

MJ and SW are employed by EVER Pharma.

### Funding

The study is sponsored by EVER Neuro Pharma.
